# Role of transcriptomic and genomic analyses in improving the comprehension of cefiderocol activity in *Acinetobacter baumannii*

**DOI:** 10.1128/msphere.00617-23

**Published:** 2023-12-11

**Authors:** Stefano Stracquadanio, Alice Nicolosi, Grete Francesca Privitera, Mariacristina Massimino, Andrea Marino, Dafne Bongiorno, Stefania Stefani

**Affiliations:** 1 Department of Biomedical and Biotechnological Sciences, Section of Microbiology, University of Catania, Catania, Italy; 2 Department of Clinical and Experimental Medicine, Unit of Math and Comp Science, University of Catania, Catania, Italy; 3 Department of Translational Research and New Technologies in Medicine and Surgery, University of Pisa, Pisa, Italy; 4 Department of Clinical and Experimental Medicine, Unit of Infectious Diseases, ARNAS Garibaldi Hospital, University of Catania, Catania, Italy; Escola Paulista de Medicina/Universidade Federal de São Paulo, São Paulo, Brazil

**Keywords:** cefiderocol, *Acinetobacter baumannii*, transcriptomic, genomic, iron transport system, antibiotic resistance

## Abstract

The mechanisms of action and resistance of cefiderocol (FDC) in *Acinetobacter baumannii* are still not fully elucidated, but iron transport systems have been evoked in its entry into the cell to reach the penicillin-binding proteins (PBPs). To capture the dynamics of gene expression related to FDC action in various conditions, we report on the genomic and transcriptomic features of seven *A. baumannii* strains with different FDC susceptibility, focusing on the variants in genes associated with β-lactam resistance and the expression of the siderophore biosynthesis and transport systems acinetobactin and baumannoferrin. We also investigated the expression of the TonB energy transduction system (ETS) and siderophore receptors *piu*A and *pir*A. The four clinical samples belonged to the same clonal complex (CC2), and the two strains with the highest FDC MICs showed peculiar variants in PBP2 and *amp*C. Similarly, the two clinical strains with the lowest MICs shared variants in an outer membrane protein as well as *amp*C. Gene expression analyses highlighted the up-regulation of the acinetobactin and baumannoferrin genes in response to iron depletion and a down-regulation in the presence of high iron concentrations. In response to FDC, gene expression seemed strain-dependent, probably due to the different metabolic features of each strain. Overall, FDC activates the ETS, confirming the active import of the drug; baumannoferrin, more than acinetobactin, appeared stimulated by FDC in an iron-depleted medium. In conclusion, iron transport systems play a clear role in the FDC uptake, and their expression likely contributes to MIC variation together with β-lactam resistance determinants.

**IMPORTANCE:**

*Acinetobacter baumannii* poses a threat to healthcare due to its ability to give difficult-to-treat infections as a consequence of our shortage of antibiotic molecules active on this multidrug-resistant bacterium. Cefiderocol (FDC) represents one of the few drugs active on *A. baumannii,* and to preserve its activity, this study explored the transcriptomic and genomic features of seven strains with varying susceptibility to FDC. Transcriptomic analyses revealed the different effects of FDC on iron transport systems, promoting mainly baumannoferrin expression—thus more likely related to FDC entry—and the energy transduction systems. These findings suggest that not all iron transport systems are equally involved in FDC entry into *A. baumannii* cells. Finally, mutations in PBPs and β-lactamases may contribute to the resistance onset. Overall, the study sheds light on the importance of iron availability and metabolic differences in FDC resistance, offering insights into understanding the evolution of resistance in *A. baumannii* strains.

## INTRODUCTION

Cefiderocol (FDC) is a siderophore cephalosporin with a peculiar mechanism of entry into bacterial cells. FDC exhibits a pyrrolidinium group in the side chain at position 3—like cefepime—and a carboxypropanoxyimino group in the side chain at position 7 of the cephem nucleus—like ceftazidime—as well as a catechol group on the side chain at position 3, conferring the ability to chelate iron and entering the cell through iron–siderophore complex channels in an energy-dependent manner (1, 2). Its antibacterial activity depends on the inhibition of cell wall synthesis, as it targets the penicillin-binding proteins (PBPs), mainly PBP3 (3). However, FDC seems able to overcome the threat of β-lactamase enzymes, especially AmpC, extended-spectrum β-lactamases (4, 5), and carbapenemase (KPC, NDM, and several OXA types), thus becoming a valid ally in the fight against carbapenem-resistant *Enterobacterales*, difficult-to-treat *Pseudomonas aeruginosa*, *Stenotrophomonas maltophilia* (6), and carbapenem-resistant *Acinetobacter baumannii* (7). The latter represents a major threat to public health (8) due to its wide antibiotic resistance, its capability to survive in the presence of disinfectants or shortage of water, and its persistence on medical devices and hospital environments.

Due to its peculiar way of entry into bacterial cells, FDC *in vitro* susceptibility testing requires iron-depleted media to mimic the Fe^3+^ starvation inside the host and, theoretically, maximize the expression of the bacterial iron transport systems responsible for the uptake of ferric iron from the extracellular environment and, thus, the carriers of the antibiotic–iron complex (9).

**Fig 1:**
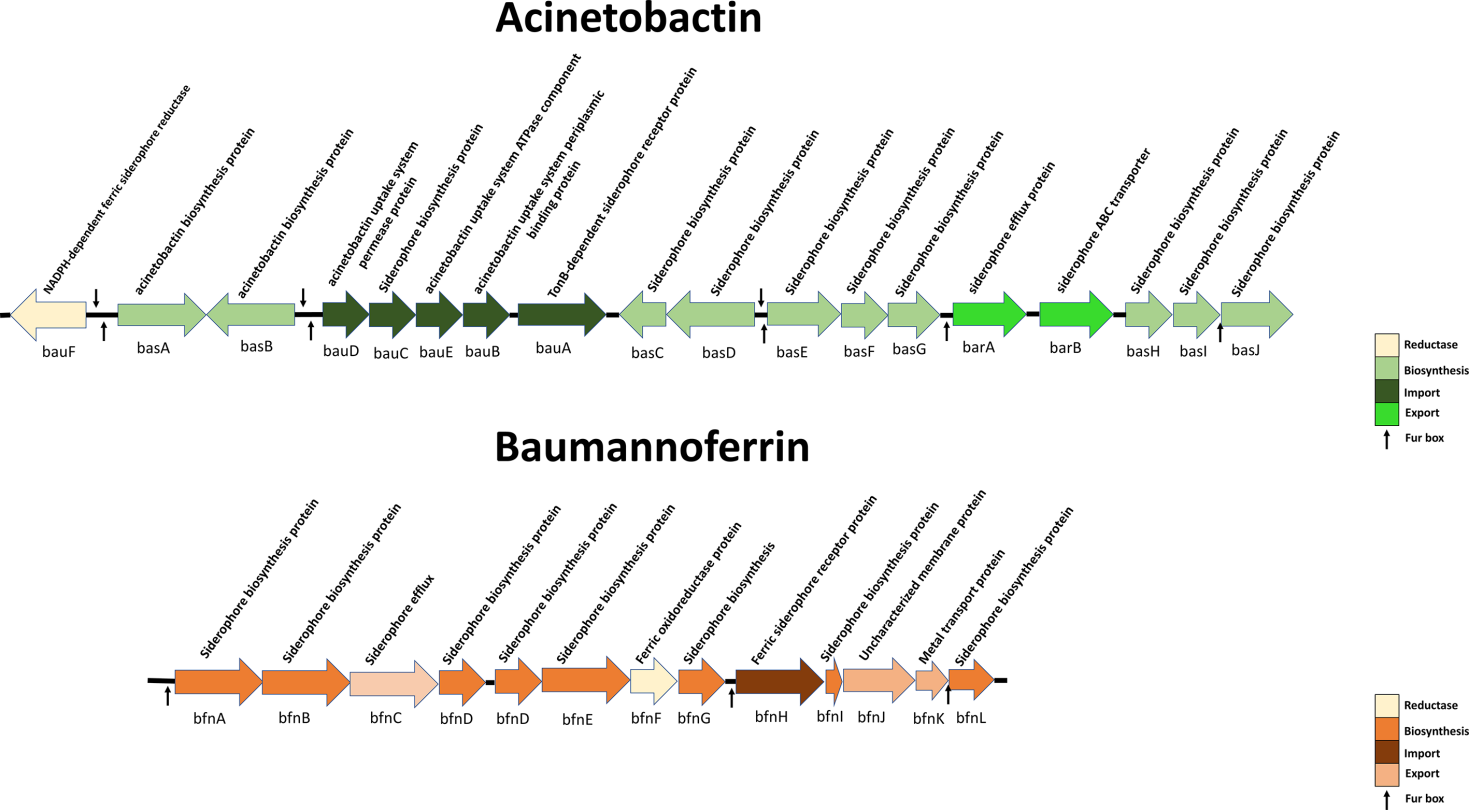
Acinetobactin and baumannoferrin gene clusters.

It is very important to shed light on bacterial features potentially related to the different responses to FDC, in order to preserve its antibacterial activity as long as possible. As the main steps of FDC action are its entry through the iron transport systems and the binding to PBPs, two of the possible mechanisms underlying FDC response may be found in mutations of the genes encoding outer membrane proteins (OMPs), PBPs and cell division proteins, siderophore receptors, and β-lactamases (10), as well as in the expression of the iron biosynthesis and transport systems. Nowadays, three major systems have been identified in *A. baumannii*: acinetobactin, baumannoferrin (Fig. 1), and fimsbactins, all of which contain genes encoding proteins for iron reduction, siderophore biosynthesis, import, and export. These systems require the TonB3/ExbB3/ExbD3 energy transduction system (ETS) to acquire the proton motive force needed for the siderophore uptake (11). Furthermore, two TonB-dependent siderophores *piu*A and *pir*A have been identified in *A. baumannii* ortholog to the same *P. aeruginosa* genes, likely playing a role in the resistance to β-lactam siderophore conjugates (12). To date, only few papers addressed the role of iron transport systems or principal FDC target gene expression, mainly through quantitative RT-PCR, suggesting the up-regulation of some iron carrier transport genes (e.g., *piu*A, *pir*A, *bau*A, and *bfn*H) as a consequence of iron starvation and subsequent FDC response, as well as the involvement of several mutations in β-lactamase genes in the development of FDC resistance (11–14).

This study primarily investigated, by transcriptomic analyses, the different expressions of iron transport systems in seven *A. baumannii* strains in three different media and in the presence or absence of FDC. The strains were varyingly susceptible to the drug. Furthermore, we genomically analyzed the role of major FDC target genes possibly related to higher MIC values.

## RESULTS

### Strains’ response to FDC

The FDC MICs of the four clinical (Abau1–Abau4) and three laboratory-adapted *A. baumannii* strains included in the study are listed in Table 1. The American Clinical & Laboratory Standard Institute (CLSI) and European Committee on Antimicrobial Susceptibility Testing (EUCAST) propose two different interpretations of FDC susceptibility testing for *A. baumannii*. CLSI indicates *S* ≤ 4 mg/L, *I* = 8 mg/L, and *R* ≥ 16 as breakpoints, while a clinical breakpoint for FDC had not yet been established by EUCAST. For this reason, our strain susceptibility categorization is not reported. However, according to the EUCAST guidelines and pharmacokinetic/pharmacodynamic (PK/PD) studies, isolates with FDC MIC > 2 in iron-depleted cation adjusted Mueller–Hinton Broth (ID-CAMHB) should be considered as non-susceptible (15). Among our isolates, two out of seven strains can be considered non-susceptible in the iron-depleted medium and three out of seven in normal CAMHB; four out of seven were resistant when Fe^3+^ was added to the medium.

**TABLE 1:** FDC MIC values at different iron concentrations^*a*^

	FDC MIC (mg/L)		
Strain	CAMHB	ID-CAMHB	ID-CAMHB+Fe^3+^
Abau1	4	8	16
Abau2	1	0.5	1
Abau3	1	4	4
Abau4	8	1	8
ACICU	8	2	4
ATCC17978	0.25	0.06	0.25
ATCC19606	0.12	0.06	0.25

^*a*^ In accordance with EUCAST, *A. baumannii* strains should be characterized as susceptible for MIC values ≤2 mg/L (*Enterobacterales*, *P. aeruginosa*, and PK/PD breakpoint). The values represent the geometric means of three replicates.

### Sequence type, clonality, and resistome

All clinical strains and ACICU belonged to the international sequence type 2 (ST2) and clonal complex (CC) 2, according to the Pasteur Institute. Therefore, they were members of the predominant clone spreading carbapenem resistance globally (16). ATCC19606 and ATCC17978 belonged to ST52 and ST77, respectively.

Resistance genes to aminoglycosides, β-lactams, macrolides, sulphonamides, and tetracyclines are listed in Table 2. Clinical strains had more resistance genes, as expected.

**TABLE 2:** Resistome of the study sample

Resistance	Gene	Abau1	Abau2	Abau3	Abau4	ACICU	ATCC17978	ATCC19606
Aminoglycosides	*aac*(3)			X				
	*aac(6′)Ib3*					X		
	*ant*(2)	X		X				
	*aadA1*			X				
	*aadA2*	X		X				
	*arm*A		X		X			
	*aph*(3′)-VIa	X		X				
	*aph*(3″)-1b		X		X			
	*aph*(6)		X		X			
β-Lactams	*bla_ADC25_*	X	X	X	X	X	X	X
	*bla*_OXA20_					X		
	*bla*_OXA23_	X	X	X				
	*bla*_OXA66_		X		X	X		
	*bla*_OXA72_				X			
	*bla*_OXA98_							X
	*bla*_OXA116_	X		X				
	*bla*_OXA259_						X	
Macrolides	*msr*E		X		X			
	*mph*E		X		X			
Sulphonamides	*sul*1	X		X	X	X		
	*sul*2						X	X
Tetracyclines	*tet*b		X		X			

### Amino acid changes in proposed FDC targets

All of the amino acid variants of the study sample—compared to the reference genome of *A. baumannii* reference strain K09-14 (susceptible to cephalosporins)—seen in the main PBPs, OMPs, and PiuA and PirA and in AmpC are listed in Table S1 and briefly reported below. The amino acid substitutions characterizing up to two strains are listed in Table 3 together with the FDC MICs of the strains and their overall gene expression in ID-CAMHB.

**TABLE 3:** Comparison of the main features of the strains with different FDC MIC values

	Amino acid changes^*e*^ (Ref. genome *A. baumannii* K09-14)								ID-CAMHB	Gene expression trend in ID-CAMHB+FDC vs ID-CAMHB FDC-free			
Strain	PBP1a	PBP1b	PBP2	PBP3	OprD^*a*^	OprD^*b*^	OprD^*c*^	AmpC	FDC MIC (mg/L)	Acinetobactin	Baumannoferrin	PiuA/PirA	ETS
Abau1			L424F		E25Q Q60H T341S K417Q	T381A F386L	F384L	P238R	8	↑	↑	↑	↑
Abau2				A514V	W243^*d*^			G247S N341T	0.5	↑	↑	↑	↑
Abau3					E25Q Q60H T341S K417Q			P238R	4	↓	↓	↓	↓
Abau4					W243			N341T	1	↑	↑	↑	↑
ACICU	L147I			A346V H370Y	K128Q L238M V299I E316D D434Y			S80R K163Q G183R N311S N379D	2	↓	↑	↑	↑
ATCC19606	V623I	V110I M726V			L236FF418I		R25W A379T	A270T	0.06	↓	↓	↑	↑
ATCC17978					M74I I72M V388L D434G	T381A F3L	F14I F18L I9L S23N		0.06	↓	↑	↑	↑

^*a*^ OprD F3P16_RS14255.

^*b*^ OprD F3P16_RS12500.

^*c*^ OprD F3P16_RS08550.

^*d*^ Indicates a stop codon.

^*e*^ Only the amino acid changes unique for one or two strains are listed as they may correlate with the different FDC responses.

#### Penicillin-binding proteins and cell division protein FtsL mutational analyses

The analyses of SNPs in the main *A. baumannii* PBPs revealed the absence of amino acid variants in PBP1a in all of the clinical strains as well as in ATCC17978, while two different SNPs were found in ACICU and ATCC19606 causing the amino acid changes L147I (17) and V623I, respectively. With regard to PBP1b, the I460V and S764P changes were common to all strains in the study, whereas P112S was found in all clinical strains and ACICU (17), and two SNPs leading to the V110I and M726V amino acid variants were seen in ATCC19606. Of note, PBP2 sequence was different only in Abau1, which carries the L424F amino acid change that may correlate with the highest MIC value of this strain. The PBP3 variants A514V and A346V/H370Y were identified in Abau2 and ACICU, respectively, as already reported for the latter (17).

FtsL did not show any sequence change in the study sample compared to the *A. baumannii* reference strain K09-14 (data not shown).

#### Outer membrane protein mutational analyses

Among the OMP encoding genes already suggested to be involved in the development of FDC resistance, CarO nucleotide sequence was different only in ACICU, not leading to amino acid changes (data not shown). Five different OprD genes, namely, F3P16_RS17620, F3P16_RS14255, F3P16_RS12500, F3P16_RS08550, and F3P16_RS01420, were identified in all the strains. The OprD F3P16_RS17620 variants L47I and T48S were found in Abau2, Abau4, and ATCC17978, the latter of which shows another 27 amino acid changes in the same protein. OprD F3P16_RS14255 was different in all of the strains in the study, with 10–16 diverse amino acid changes in each strain. Interestingly, Abau1 and Abau3 shared the same amino acid variants, similarly as Abau2 and Abau4—both with a W243* stop codon resulting in a truncated protein (243 aa instead of 448 aa)—while ACICU was the only strain having K128Q, L238M, V299I, E316D, and D434Y. The L236F and F418I amino acid changes were unique for ATCC19606 OprD F3P16_RS14255, while ATCC17978 was the only strain showing the I172M, V388L, and D434G variants in the same gene. With regard to OprD F3P16_RS12500, the same four amino acid changes were common to Abau2, Abau3, Abau4, and ACICU, whereas ATCC19606 only had two of them. In the same gene, Abau1 presented two more amino acid changes, one of which was unique for this strain (F386L) and the other one (T381A) shared with ATCC17978, the latter also having a unique F3L variation. When analyzing OprD F3P16_RS08550, it was evident that all strains but the two ATCCs shared the same eight amino acid variants. Once again, of note, Abau1 had a supplementary modification in the same gene, F384L. ATCC19606 OprD F3P16_RS08550 exhibited seven out of the eight variants, as it lacked F14V but had the unique A379T. ATCC17978 was characterized by four peculiar variants at the beginning of its OprD F3P16_RS08550 protein (F14I, F18L, I19L, and S23N) next to six of the amino acid changes reported for the other strains.

#### TonB-dependent siderophore receptors and ETS gene mutational analyses

PiuA and PirA, as well as TonB3, ExbB3, and ExbD3, were found to exhibit the same amino acid sequences as the *A. baumannii* reference strain K09-14 throughout the sample (data not shown).

#### Class C β-lactamase mutational analyses

The AmpC amino acid sequence of ATCC17978 was identical to that of *A. baumannii* K09-14 AmpC, while all other strains shared the V119E variant. All clinical strains also shared the Q150K, P167S, and F283R amino acid changes. Both Abau1 and Abau3 had the P238R modification; similarly, Abau2 and Abau4 shared the N341T amino acid change, which was missing in the other strains. Moreover, Abau2 was also characterized by the presence of the G247S variant. Finally, ACICU was different from the clinical strains, having the unique S80R, K163Q, G183R, N311S, and N379D variants, whereas ATCC19606 was characterized by the A270T, besides the shared V119E and F283R amino acid changes (Table S1).

### Iron transport and biosynthesis system expression

A differential gene expression (DGE) analysis of transcriptomic data has shown a great number of differently expressed genes related to several bacterial metabolic pathways (e.g., amino acid metabolism, glucose metabolism, energetic metabolism, and fimbriae production). To assess the impact of diverse iron and FDC concentrations on *A. baumannii* with different FDC susceptibility, here, we report the DGE analyses for the main *A. baumannii* iron transport and biosynthesis systems, highly involved in the uptake of FDC. Of note, of the three systems present in Acinetobacter, our strains had only two of them, i.e., acinetobactin and baumannoferrin; the fimsbactin system was only found in the genome of ATCC17978 and was, therefore, excluded from the analyses.

**Fig 2:**
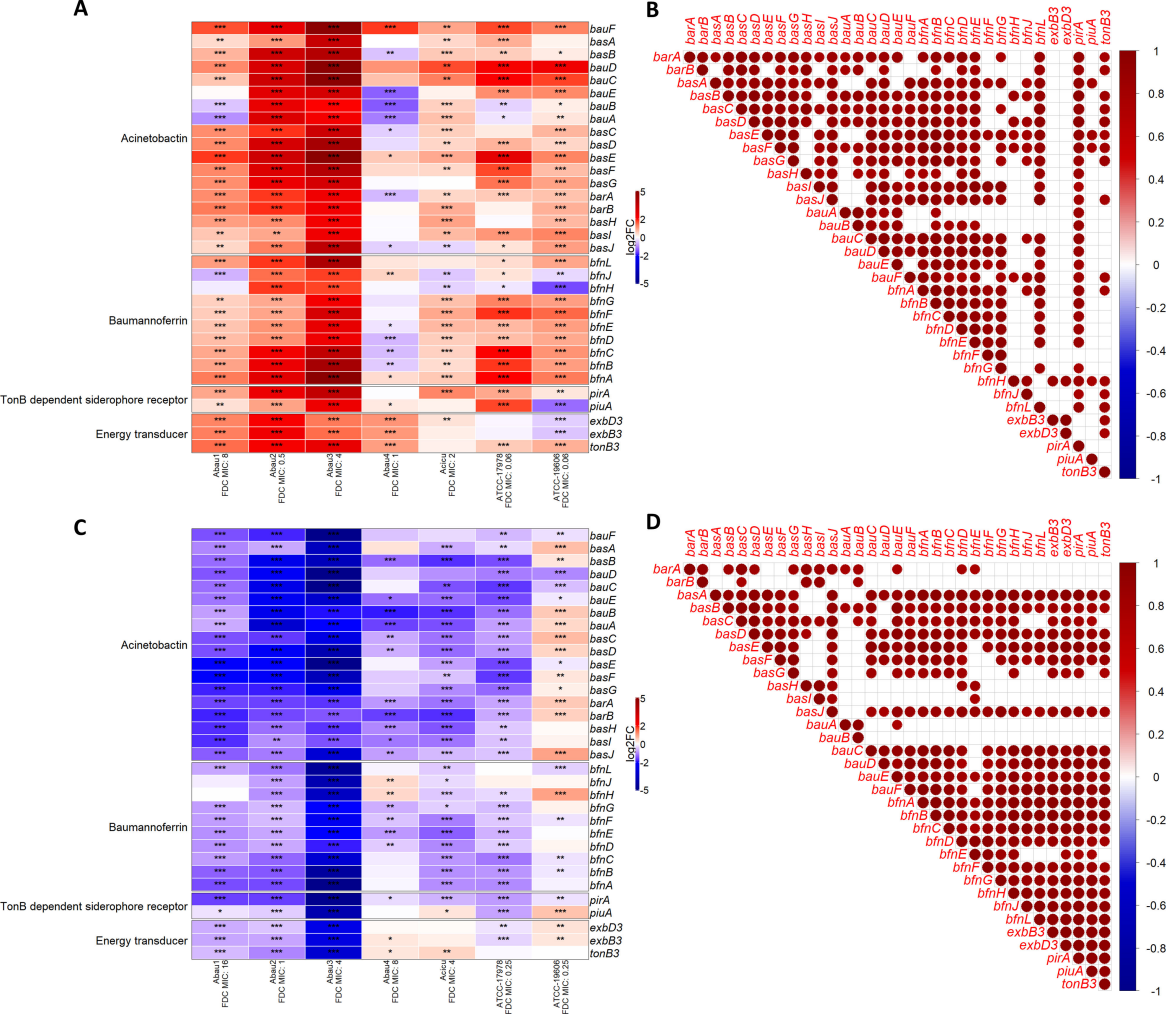
Effect of different iron concentrations on the expression of iron biosynthesis and transport system genes (**A and B**) ID-CAMHB vs CAMHB and (**C and D**) ID-CAMHB+Fe^3+^ vs ID-CAMHB. Heatmaps in A and C report the different expressions of the genes in the study with * indicating the probability of the different gene expression (PDE) being statistically significant (*0.85 ≤ PDE < 0.90; **0.90 ≤ PDE < 0.95; ***PDE ≥ 0.95). In B and D, the dots show the results of Pearson’s correlation analysis. The statistically significant (*P*-value ≤0.05) positive (red) or negative (blue) correlation of gene expressions indicates a clustered trend in at least four out of seven strains.

**Fig 3:**
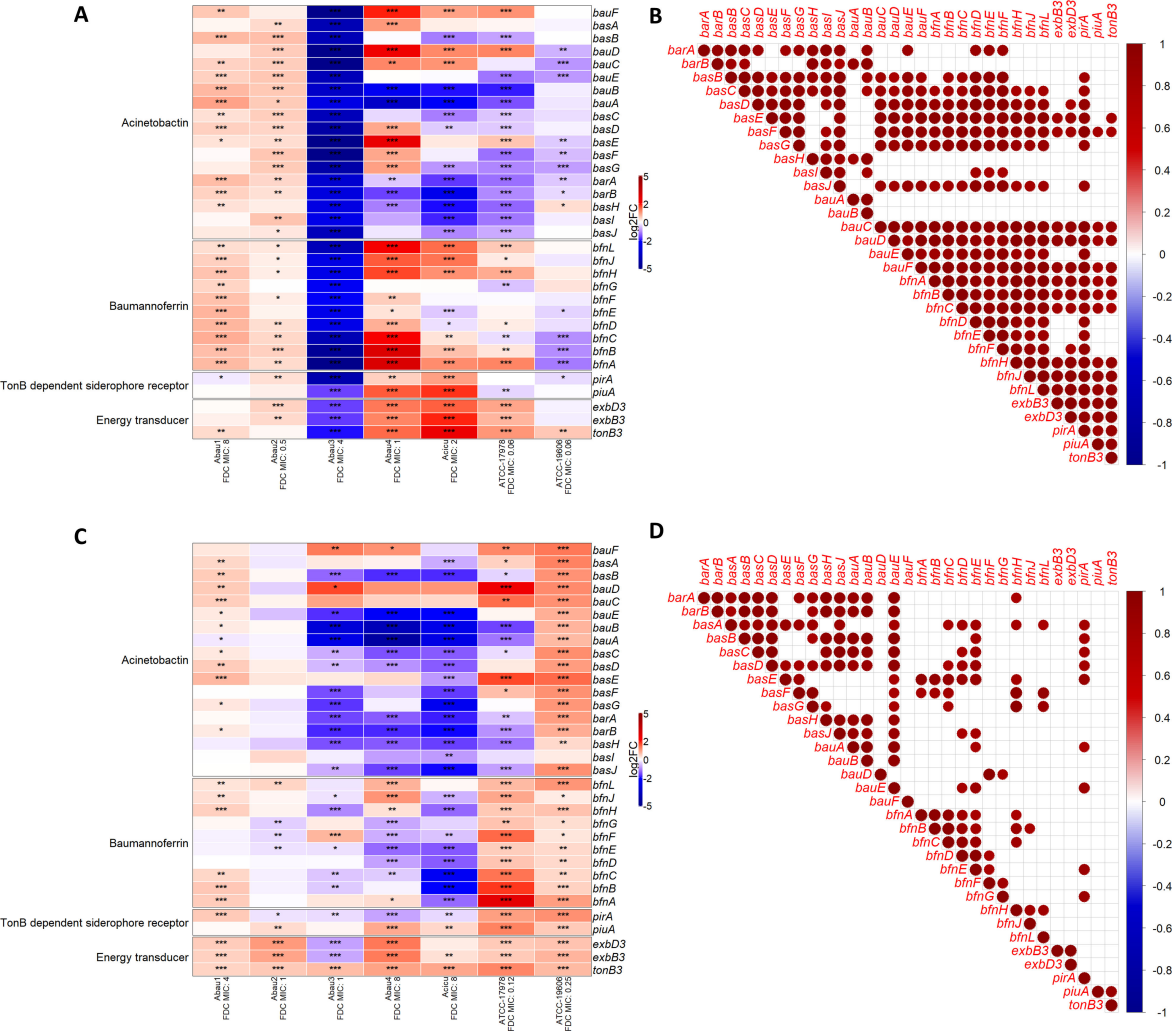
Effect of FDC sub-inhibitory concentrations on the expression of iron biosynthesis and transport system genes (**A and B**) ID-CAMHB+FDC vs ID-CAMHB FDC-free and (**C and D**) CAMHB+FDC vs CAMHB FDC-free. Heatmaps in A and C report the different expressions of the genes in the study with * indicating the probability of the different gene expressions being statistically significant (*0.85 ≤ PDE < 0.90; **0.90 ≤ PDE < 0.95; ***PDE ≥ 0.95). In B and D, the dots show the results of Pearson’s correlation analysis. The statistically significant (*P*-value ≤0.05) positive (red) or negative (blue) correlation of the gene expressions indicates a clustered trend in at least four out of seven strains.

Acinetobactin and baumannoferrin, as well as *piu*A and *pir*A, and ETS *exb*B3, *exb*D3, and *ton*B3 varied in gene expression depending on the different culture conditions and in response to the different iron concentrations and exposition to FDC at two sub-MIC concentrations, as reported in Fig. 2 and 3 and described below.

In particular, the effect of different iron concentrations on the expression of the genes involved in iron biosynthesis and transport is reported in Fig. 2A through D and described below.

#### ID-CAMHB FDC-free vs CAMHB FDC-free

The expression analysis of the genes involved in siderophore synthesis and transport when Fe^3+^ concentration is <1/20 of the concentration in the normal CAMHB medium highlighted a strong up-regulation of all acinetobactin and baumannoferrin genes as well as *piu*A, *pir*A, and ETS genes, mainly in Abau2 and Abau3. Conversely, less than half of the statistically differentially expressed genes(DEGs) were up-regulated (8/18), while 11/18 genes were down-regulated in Abau4. With regard to Abau1, ACICU, and the two ATCCs, the acinetobactin and baumannoferrin genes were globally up-regulated; only two to five genes were down-regulated. Concerning acinetobactin, *bau*A and *bau*B (import genes) were down-regulated in Abau1 and ATCC17978, whereas *bas*J (biosynthesis gene) was down-regulated in ACICU. Baumannoferrin genes *bfn*J (export) and *bfn*H (import) were both down-regulated in ACICU and ATCC19606, whereas *bfn*J was the only down-regulated gene within the baumannoferrin cluster in Abau1, and *bfn*H was down-regulated in ATCC17978. Of note, ATCC19606 was the only strain showing down-regulation of *exb*B3 and *exb*D3 even in the presence of up-regulated *ton*B3 (Fig. 2A).

The overall increased expression trend in the iron-depleted growth medium was observed with both acinetobactin and baumannoferrin, as well as with the other TonB-dependent siderophore receptors and ETS.

Correlation analyses consistently showed that almost all acinetobactin and baumannoferrin genes had the same trend in response to iron depletion (less evident for *bau*A, *bfn*H, and *bfn*J). Furthermore, *pir*A expression showed greater concordance with other genes than *piu*A. Finally, among the ETS genes, only *ton*B3 had a significant expression trend similar to that of some of the siderophore biosynthesis and transport systems, mainly paired with export (*bar*A, *bar*B, and *bfn*J) and biosynthesis (*bas*A-*bas*G, *bas*J, and *bfn*A) genes (Fig. 2B).

#### ID-CAMHB+Fe^3+^ FDC-free vs ID-CAMHB FDC-free

As expected, we found a consistent down-regulation of siderophore biosynthesis and transport system genes when Fe^3+^ concentration was 40 times higher than in iron-depleted conditions, as shown in Fig. 2C. All genes were statistically down-regulated in Abau2 and Abau3; there were only significant down-regulation or no expression changes in Abau1 and ATCC17978. Abau4, instead, had two up-regulated genes within the baumannoferrin system and ETS. With regard to ACICU, this only showed up-regulation for *piu*A and *ton*B3. ATCC19606 was characterized by a different behavior; in fact, 11/18 genes of the acinetobactin system were statistically up-regulated, and 5/18 genes were down-regulated (*bau*F, *bau*D, *bau*C, *bau*E, and *bas*E), while only one gene in the baumannoferrin system was up-regulated (*bfn*H), as were *piu*A, *exb*D3, and *exb*B3.

Similar expression changes were highlighted in almost all acinetobactin and baumannoferrin genes, as well as between them and *piu*A, *pir*A, *exb*B3, *exb*D3, and *ton*B3 (Fig. 2D). This trend was less evident for three iron biosynthesis (*bas*H, *bas*I*,* and *bfn*E) and two iron import (*bau*A and *bau*B) genes and, to a lesser extent, also for the export genes *bar*A and *bar*B. Interestingly, all these genes but *bfn*E belonged to the acinetobactin cluster, the latter being less compliant with the expected results.

The effect on DGE of adding FDC at sub-inhibitory concentrations is reported in Fig. 3A through D and analyzed in the following paragraphs.

#### ID-CAMHB+FDC vs ID-CAMHB FDC-free

The siderophore gene expression in the presence of FDC at infinitesimal iron concentrations, widely indicated as the condition maximizing FDC entry, highlighted a non-uniform strain-dependent response of the analyzed clusters of genes. As reported in Fig. 3A, acinetobactin, baumannoferrin, and ETS genes were up-regulated in Abau1 and Abau2, while *pir*A was slightly down-regulated in Abau1 and up-regulated in Abau2. Conversely, *piu*A expression did not vary in Abau1 and was not significantly up-regulated in Abau2. Abau3 was characterized by a strong down-regulation of all analyzed genes. Acinetobactin genes showed an increasing “trend” toward down-regulation, more evident when moving from Abau4 to ACICU and from ACICU to the two ATCCs. Baumannoferrin genes, *piu*A and *pir*A, and energy transducers were all up-regulated in Abau4. A similar behavior characterized ACICU and ATCC17978, with the exception of two down-regulated genes within the baumannoferrin system (*bfn*E and *bfn*D biosynthesis genes in ACICU, *bfn*C and *bfn*G export and biosynthesis genes in ATCC17978) and the down-regulation of *piu*A in ATCC17978. Conversely, ATCC19606 showed a down-regulation of the baumannoferrin genes (similar to its acinetobactin genes) and *pir*A and up-regulation of *ton*B3.

FDC addition, with the only exception of the non-susceptible Abau3, activated all genes related to the TonB3 ETS, demonstrating the expression of these genes more than others.

The clustered comparison of gene expression in Fig. 3B highlighted an overall equal expression trend between the acinetobactin and baumannoferrin genes; however, *bar*A, *bar*B, *bas*H, *bau*A, and *bau*B expression mainly correlated with other acinetobactin genes. *pir*A and *piu*A showed different trends, the latter being related to fewer acinetobactin genes (only the *bas*F iron biosynthesis gene, two import genes *bau*C and *bau*D, and *bau*F reductase), while *pir*A expression was in line with almost all of the acinetobactin iron biosynthesis genes (i.e., *bas*B*–bas*G and *bas*J), *bau*C*–bau*E import genes, and the reductase gene *bau*F. The ETS genes had the same expression trend among them and as the iron import and biosynthesis genes of the acinetobactin and the different components of the baumannoferrin system.

#### CAMHB+FDC vs CAMHB FDC-free

As for the previous comparison, the addition of sub-inhibitory concentrations of FDC to CAMHB highlighted different strain-related changes in the iron biosynthesis and transport system gene expression (Fig. 3C). Abau1 was still only characterized by statistically significant up-regulation of all genes but *bau*A, although their expression was less important. The acinetobactin genes in Abau2 did not show any statistically significant difference in expression. Meanwhile, of the baumannoferrin genes, three were down-regulated (biosynthesis *bfn*G and *bfn*E, reductase *bfn*F) and one up-regulated (biosynthesis *bfn*L). Still considering Abau2, *pir*A and *piu*A had opposite expressions: the former was down-regulated and the latter up-regulated, while all ETS genes were up-regulated. Abau3, Abau4, and ACICU were characterized by the down-regulation of the acinetobactin and baumannoferrin genes (more evident in ACICU than in the other two strains), down-regulation of *pir*A, and up-regulation of *piu*A (not significant in Abau3). A similar concordance was not found in the ETS genes: in fact, while *ton*B3 was up-regulated in all three strains, *exb*B3 and *exb*D3 were down-regulated in Abau3 and up-regulated in Abau4 and ACICU.

Once again, FDC addition seems to be effective in up-regulating the TonB3/ExbB3/ExbD3 energy transducers in all strains, with the exception of the non-susceptible Abau3. The acinetobactin genes in ATCC17978 did not have a univocal expression trend (with 6/18 genes significantly up-regulated and 8/18 genes significantly down-regulated), whereas all baumannoferrin, TonB-dependent siderophore receptors, and ETS genes were up-regulated. This up-regulation trend was more evident in ATCC19606, characterized by the up-regulation of all analyzed genes (with the exception of acinetobactin biosynthesis gene *bas*I, which was not statistically significantly up-regulated).

Correlation analyses (Fig. 3D) confirmed a strain-dependent gene expression. Indeed, no correlation was seen in this comparison in the expression of the acinetobactin and baumannoferrin genes, and gene expressions were also more discordant within the two systems than in the previous analyses. *pir*A expression paired with iron import (*bauA*, *bau*E) and biosynthesis acinetobactin (*bas*A*–bas*E) genes, while *piu*A only showed concordant expression with *ton*B3, but not with either the acinetobactin or baumannoferrin genes. With regard to the ETS genes, only the expression of *exb*B3 and *exb*D3 was correlated, but so was not with the other iron transport and biosynthesis system genes.

### Correlations between FDC MIC values and gene expression in different growth media

**Fig 4:**
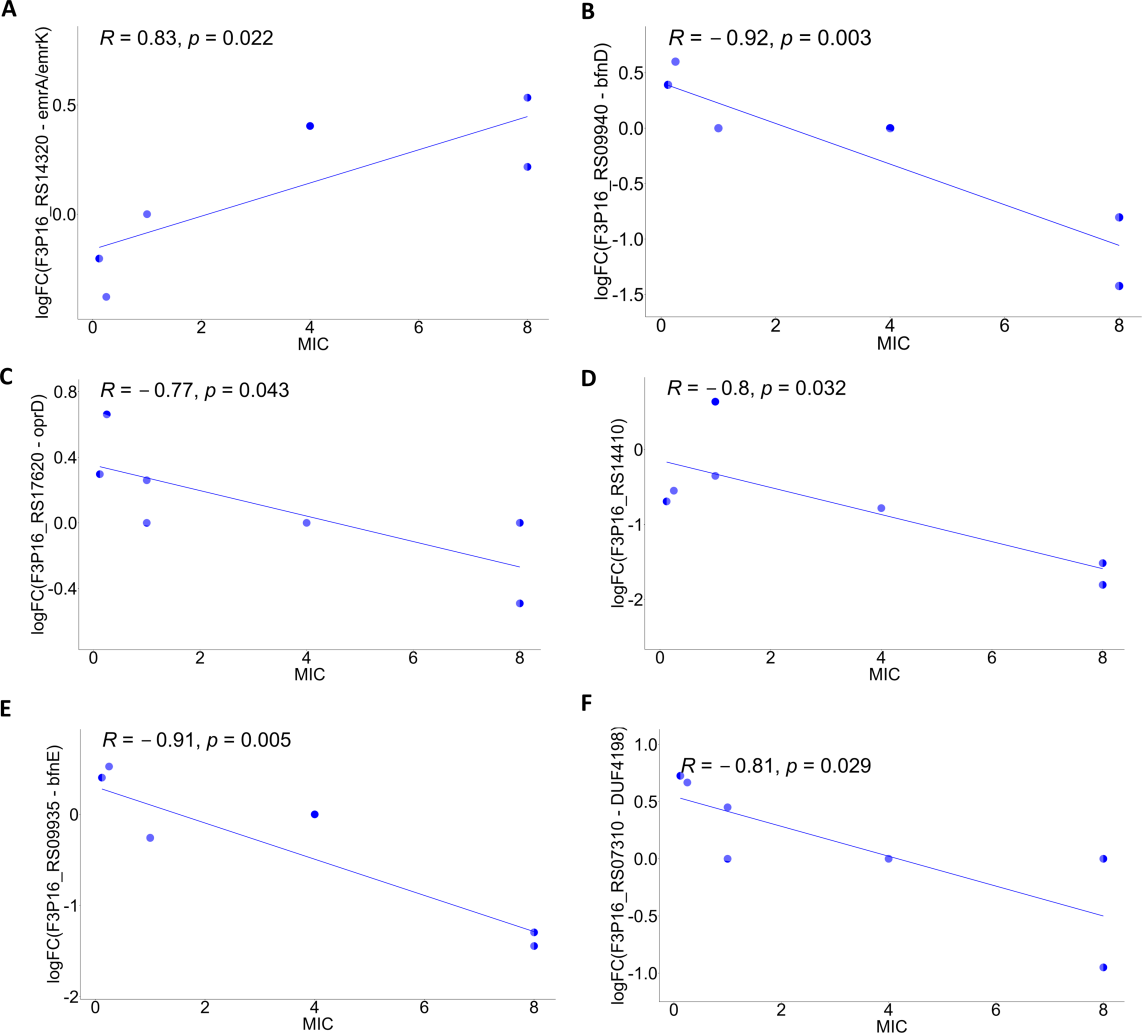
Scatter plots of the correlation between FDC MIC variations and iron or drug resistance-related gene expression in CAMHB. Blue dots indicated the FDC MIC of the strains in CAMHB. (A) *emr*A/*emr*K multidrug export protein gene. (B) *bfn*D siderophore biosynthesis gene. (C) *opr*D porin gene. (D) bacteriohemerythrin gene. (E) *bfn*E siderophore biosynthesis gene (*iucA*/*iuc*C family). (F) DUF4198 metal transport family protein gene.

The analysis of correlation showed an association between MIC values and several genes as reported in Tables S2 and S3. Only the expression trends of genes likely related to antibiotic resistance have been graphically represented in the scatter plots as shown in Fig. 4. To note, just the addition of FDC to normal CAMHB displayed correlations in siderophore (i.e., DUF4198 domain-containing protein, IucA/IucC, and AcsC), iron-dependent oxygen transporter (bacteriohemerythrin), efflux pump (EmrA/EmrK), and porin genes (OprD), while in ID-CAMHB, the analyses revealed that only the expression of metabolic, tRNA, and ATP-dependent system genes correlated with MIC variation.

Interestingly, the expression of the genes for siderophores, iron-dependent oxygen transporter, and porin negatively correlated with the increase of the MIC values (the more resistant was the strain, the less expressed were the genes), while the expression of the efflux pump gene increased with the increasing of the FDC MIC values (positive correlation).

## DISCUSSION

*A. baumannii* is a resilient pathogen that relies on various cellular processes to adapt to challenging environments, including within the host, and to selective antibiotic pressure. With an increasing number of multidrug-resistant (MDR) strains of *A. baumannii* emerging, elucidating their regulatory mechanisms and direct regulons can provide new information on the mechanisms of action and resistance to identify promising targets for novel therapeutic interventions.

FDC is one of the drugs showing efficacy against this troubling MDR species; it acts like a Trojan horse, mimicking the structure of siderophores. When FDC binds to iron, it forms a stable complex that is recognized by bacterial iron transporters. These transporters then take the FDC–iron complex into the bacterial cell, where it can exert its antimicrobial activity. Therefore, iron concentration at FDC entry is crucial for its mechanism of action (9).

Herein, we have explored how FDC affects the expression of the main iron transport systems in some *A. baumannii* isolates with different FDC MICs, at various iron concentrations. The study was performed through the novel approaches of whole-genome sequencing (WGS) and RNA-seq techniques. In particular, transcriptomics, coupled with WGS for the identification of mutations likely to confer antibiotic resistance, is a powerful tool for studying this phenomenon. It allows for the identification of changes in gene expression patterns that occur in response to antibiotic exposure as well as helps identify genes that are responsible for antibiotic resistance by comparing the transcriptome of resistant and susceptible bacteria (18–23).

According to our genomic analyses, all our clinical strains belonged to the CC2 international clone and carried a plethora of antibiotic resistance genes, especially for aminoglycosides, β-lactams, macrolides, sulphonamides, and tetracyclines. Clinical strains probably had more chances to acquire new resistance determinants as shown in our results. Genomic analyses somewhat confirmed the differences reported in the evaluation of the proposed FDC target mutations as well as the different susceptibility to FDC of the clinical strains. The analyses of the mutations in these genes suggested that they may be involved in cephalosporins and/or FDC resistance, as already described (24–31). Among the main PBPs, only PBP2 may have an amino acid change related to FDC resistance (L424F), as it is only harbored by Abau1 (the strain with the highest MIC). Amino acid changes in the other PBPs are shared by or unique for susceptible strains, for which reason they should be considered less significant. The identified OprD gathered multiple variants, but—once again—only Abau1 had peculiar variants in two of them, while another porin was characterized by four amino acid changes shared by both FDC non-susceptible strains Abau1 and Abau3. Notwithstanding, these variants are more likely attributable to a generic β-lactam resistance, as these OMPs are less involved in FDC entry. Interestingly, the two clinical strains with the lowest FDC MICs were characterized by a truncated OprD protein, halved compared to the original one, thus probably not properly working. Their low MIC values led to the hypothesis that the missing function of one of these proteins may be counterbalanced by the presence of cognate proteins and that FDC does not mainly enter through these channels. With regard to AmpC, the most important amino acid change seems to be P238R, as this is common to both FDC non-susceptible strains. However, AmpC sequence was different in all other strains but ATCC17978 when compared to the cephalosporin-susceptible *A. baumannii* K09-14. ACICU, on the other hand, was characterized by a plethora of amino acid changes, and albeit susceptible to FDC, its MIC value was higher than that for the ATCCs and the other two susceptible isolates.

Our results would lead to considering the complexity and multiplicity of factors involved in FDC testing and determination of resistance. First off, we confirmed the importance of Fe^3+^ in the regulation of the main iron biosynthesis and transport systems of *A. baumannii*, predominantly acinetobactin and baumanoferrin in our strains. The use of the ID-CAMHB, evaluated in comparison with CAMHB, showed the up-regulation of all genes clustered in the two systems, and this observation was significant for almost all isolates. The lack of iron in the medium varyingly affected the transcription of ETS genes, demonstrating a sort of intra-species variability, probably linked to the different energy status of the microorganism. The addition of a high concentration of Fe^3+^ to the medium changed the scenario completely: in all strains—with the exception of ATCC19606—repression of gene expression was observed in all systems, including energy transducers. This generalized and statistically significant behavior showed some specific variability in genes related to the ferric reductase and import genes such as *bas*H, *bas*I, and *bas*J of the acinetobactin cluster, as well as the *bau*A and *bau*B genes related to iron import, in which repression was less represented.

Comparing the effect of sub-inhibitory FDC supplementation in the different culture conditions (i.e., ID-CAMHB vs CAMHB, as high iron concentrations are allegedly detrimental for FDC testing), an overall greater expression of the iron uptake clusters was observed in ID-CAMHB compared to a condition in which Fe^3+^ was 20 times more concentrated (CAMHB). These expected results were statistically confirmed in Pearson’s correlation analysis, in which the same DEGs were associated in at least four out of the seven strains included.

Sub-inhibitory concentrations of FDC in ID-CAMHB were seen to be active in promoting a positive expression trend of the acinetobactin and baumannoferrin genes, which may potentially increase the entry of the antibiotic into bacterial cells, as suggested by the use of ID-CAMHB in MIC testing. Abau3 was characterized by a stronger differential expression of all analyzed genes in all culture conditions. An evident down-regulation trend was also observed for Abau3 in the presence of FDC in ID-CAMHB, unlike for the other clinical strains. With the exception of Abau3, the addition of FDC to ID-CAMHB stimulated the expression of the ETS, suggesting the occurrence of active transport of FDC inside the cell, as described for the Fe-siderophore system entry (32). Interestingly, not only was the ETS activated by iron depletion, but also the addition of FDC to a medium with standard iron concentrations (CAMHB) promoted its expression, whose genes were only partially repressed in the presence of high iron concentrations. Finally, FDC seems to have different effects on acinetobactin and baumannoferrin, enhancing the expression of the genes of the latter cluster and impairing acinetobactin gene expression.

Overall, our data demonstrated that not all iron-chelating systems are present and used in *A. baumannii* isolates and that iron depletion is a key factor for implementing FDC entry and, consequently, is of importance for *in vitro* testing of FDC. These results are in accordance with previous studies dealing with the importance of iron depletion in the testing conditions for FDC (33–35).

Furthermore, our isolates showed varying degrees of susceptibility to FDC (range 0.5–16 mg/L), and these increased MIC values cannot be attributed to a single mechanism regulating the entry of the drug into the cell but rather to a complex network of events also related to the accumulation of mutations in important antibiotic targets such as PBP2, AmpC, and OMP.

FDC, in the ID medium, also affects all iron uptake transport and energy activation systems in a strain-related manner but affects primarily the ETS that seems to be required for drug entry. These results, which involve the molecular motor as the first and common response to FDC, suggested the role played by the metabolic complexity of *A. baumannii* as well as several of its characteristics in making this species resistant to various antibiotics and adverse environments.

Analyzing the variation of the expression of the whole transcriptome and the different FDC MIC values, we found that the most relevant associations between MIC and genes related to iron acquisition or utilization as well as porins and efflux pumps occurred only when FDC was added to a growth medium with average iron levels. The positive correlation between MIC and the expression of few metabolic genes observed when FDC was added to a medium with very low concentration of iron could be a consequence of the minor effect of the drug on FDC non-susceptible strains.

In conclusion, our study, which was performed to define (i) the role of different iron concentrations (high and low concentrations) on FDC entry and activity in our isolates and (ii) the correlation of these different gene expressions and FDC MIC values with mutations in the most important targets responsible for resistance, highlighted some different issues in these complex mechanisms.

The complete transcriptomics of all isolates highlighted the presence of more than 800 differently expressed genes for each tested condition, related to various aspects of bacterial metabolism. As the aim of our research was to investigate the peculiar features of *A. baumannii* strains with different susceptibility to cefiderocol and their response to diverse iron concentrations and FDC, we focused on possible FDC targets and iron–drug uptake systems. This aspect, together with the limited number of strains included, may represent a limitation of the study. Further analysis would need to be performed to better understand possible predictive markers involved in the progression toward a more resistant phenotype.

## MATERIALS AND METHODS

### Bacterial strains, FDC MIC determination, and culture conditions

The seven *A. baumannii* strains included in the study—Abau1–Abau4, ACICU, ATCC17978, and ATCC19606—were already characterized for their ST, Oxa genes, MEM, and COL resistance as well as their response to FDC in a previously published study (36). The clinical samples (Abau1–Abau4) are representative of different geographic origins and years of isolation: Abau1 and Abau2 were isolated at the “Azienda Ospedaliero Universitaria Policlinico G.Rodolico–San Marco” (Catania, Italy) in 2020; Abau3 was retrieved at the “Policlinico Sant’Orsola” (Bologna, Italy) in 2014; Abau4 was collected at the “Azienda Ospedaliero–Universitaria Pisana” (Pisa, Italy) in 2016.

To investigate the effect of iron concentration in the medium on iron transport gene expression, the same lot of CAMHB (Becton, Dickinson and Company, Maryland, USA) was used to prepare three different culture media with various iron concentrations: CAMHB (Fe^3+^ 0.084 µg/mL), iron-depleted CAMHB (Fe^3+^ 0.005 µg/mL), and ID-CAMHB+Fe^3+^ (Fe^3+^ 0.200 µg/mL). Iron depletion was performed using the Chelex 100 resin (Bio-Rad Laboratories, California, USA), as reported by Hackel et al. (35); ID-CAMHB+Fe^3+^ was prepared by adding FeCl_3_ to ID-CAMHB with the aim of obtaining a medium with twice the Fe^3+^ concentration of CAMHB. The iron concentration of each media was assessed using the Iron Assay Kit (cat. no. MAK025-1KY, Merck/Sigma-Aldrich, Germany) according to the manufacturer’s protocol.

FDC minimum inhibitory concentration (MIC) in the different conditions was assessed as previously published (36).

In order to study the influence exerted by FDC on iron transport gene expression, all strains were cultured in the absence and presence of two sub-MIC concentrations of antibiotic in the following different growth conditions: (i) CAMHB FDC-free and (ii) CAMHB+FDC; (iii) ID-CAMHB FDC-free and (iv) ID-CAMHB+FDC; and (v) ID-CAMHB+Fe^3+^ FDC-free.

For each culture condition, the strains were grown in 30 mL of broth (250-mL flasks, normal air) starting from a 1:100 dilution of overnight culture in CAMHB. Flasks were incubated at 37°C with agitation (150 rpm) until the exponential growth phase (OD_450_: 0.45–0.55).

The bacterial pellet was recovered by centrifugation at 4,000 rpm for 10 min at 4°C, resuspended in RNAprotect Bacteria buffer (cat. no. 76506, QIAGEN, Germany), transferred into sterile microcentrifuge tubes, and stored at –20°C pending nucleic acid extraction.

### Genome extraction and sequencing

#### DNA extraction

DNA extraction was carried out with the QIAamp DNA minikit (product 51304; QIAGEN, Hilden, Germany) following the manufacturer’s protocol. DNA was quantified using an Eppendorf BioPhotometer D30 and the Qubit fluorimeter double-stranded DNA broad-range assay kit (product 32850; Invitrogen, Carlsbad, CA, USA).

#### Whole-genome sequencing

WGS was performed on a MiSeq platform as already published (36) and according to the manufacturer’s instructions provided in the Illumina DNA preparation (M)-tagmentation (24 samples) for the Illumina kit (product 20018707; Illumina, Inc., San Diego, CA, USA) (37, 38).

### RNA extraction and reverse transcription

RNA extraction was performed using the RNeasy Mini Kit (cat. no. 74104, QIAGEN, Germany) according to the manufacturer’s protocol. RNA was quantified using the Qubit RNA High Sensitivity kit (cat. no. Q32852, Thermo Fisher Scientific, Massachusetts, USA) on Qubit 3.0 Fluorometer (cat. no. Q33216, Thermo Fisher Scientific, Massachusetts, USA) and reverse-transcribed using the QuantiTect Rev. Transcription Kit (cat. no. 205311, QIAGEN, Germany) at a cDNA concentration of 50 ng/µL.

### RNA sequencing

RNA-seq was performed by Genomix4Life S.r.l. (Baronissi, Salerno, Italy). RNA concentrations were determined by a NanoDrop One spectrophotometer (Thermo Fisher), and its quality was assessed with TapeStation 4200 (Agilent Technologies). Indexed libraries were prepared from 150 ng/ea purified RNA with Stranded Total RNA Prep with Ribo-Zero Plus (Illumina) for ribosomal RNA depletion according to the manufacturer’s instructions. Libraries were quantified using TapeStation 4200 (Agilent Technologies) and a Qubit fluorometer, then pooled in such a way that each index-tagged sample was present in equimolar amounts. The pooled samples were subjected to cluster generation and sequencing using an Illumina NovaSeq6000 System (Illumina) in a 2 × 100 paired-end format.

The raw sequence files generated (.fastq files) underwent quality control analysis using FastQC (http://www.bioinformatics.babraham.ac.uk/projects/fastqc).

### Bioinformatic and statistical analyses

Raw paired-end sequence files were aligned using hisat2 (39) employing *A. baumannii* strain K09-14 (NZ_CP043953.1) as the reference genome. Thereafter, FeatureCounts (v2.0.1) was used to count mapped reads for genomic features using the paired-end mode and “gene” as a feature for the count. Subsequently, the raw count file was analyzed using R (v.4.2.1) package NOISeq (40) to simulate technical replicates using default parameters and obtain DEGs between condition pairs. DEGs were only considered significant if their probability was greater than 0.85. Iron-associated genes were represented by heatmaps employing the ComplexHeatmap (v.2.12.1) package (41) using an asterisk as a significant symbol to indicate the probability of differential expression: *0.85 ≤ PDE < 0.90; **0.90 ≤ PDE < 0.95; ***PDE ≥ 0.95. To reveal the trends of expression of the acinetobactin, baumannoferrin, TonB3-ETS (*ton*B3, *exb*B3, and *exb*D3), and *piu*A and *pir*A, Pearson’s correlation analyses were performed. Genes were only retained for correlation analysis if their logFC (log of the fold change) was significant in more than half (no. 4) of the strains considered in this work. Results were plotted using the corrplot R package (42), where 1 stands for a full positive correlation and −1 indicates a full negative correlation, whereby only correlations with a *P*-value ≤0.05 were considered as significant. Moreover, we also compared the gene expression with MIC in CAMHB+FDC vs CAMHB FDC-free and ID-CAMHB+FDC vs ID-CAMHB FDC-free conditions. This analysis was plotted using a scatter plot with the package ggpubr (v.0.6.0) (43). Furthermore, we analyzed the mutations of genes likely involved in FDC resistance such as PBPs, *fts*L, *car*O, *opr*D, *piu*A, *pir*A, and *amp*C (10, 12, 26). ACICU (NC_010611.1) and ATCC19606 (AP022836) FASTA and GenBank files were downloaded from NCBI (44). The paired-end raw reads of Abau1, Abau2, Abau3, Abau4, and ATCC17978 were analyzed employing BacPipe (45) with built-in parameters, using *A. baumannii* K09-14 (NZ_CP043953.1) as reference. Moreover, to identify punctual mutations, the output assemblies from BacPipe for each sample were bwa-aligned using PBPs, *fts*L, *car*O, *opr*D, *piu*A, *pir*A, and *amp*C gene sequences as references (46). Finally, files were sorted using SAMtools, variants were called, and consensus sequence was generated using BCFtools (47, 48). Amino acid replacement was inspected using Transeq (49) and BLASTp (50). Additionally, antibiotic resistance genes were examined in depth with both BacPipe [CARD (51) and ResFinder (52) database] and AMRfinder (53) to obtain the resistome profile of our strains.

For ST attribution of the strains, their genomes were analyzed with the PubMLST online tool (54).

## Data Availability

Transcriptomic and genomic data are available on SRA under the following accession numbers: PRJNA850903 and PRJNA985120.

## SUPPLEMENTAL MATERIAL

Legends msphere.00617-23-s0001.docx Legends for the supplemental materials.

Table S1 msphere.00617-23-s0002.docx Amino acid changes in proposed determinant of FDC resistance.

Table S2 msphere.00617-23-s0003.xlsx MIC-gene expression correlations in CAMHB.

Table S3 msphere.00617-23-s0004.xlsx MIC-gene expression correlations in IDCAMHB.

ASM does not own the copyrights to Supplemental Material that may be linked to, or accessed through, an article. The authors have granted ASM a non-exclusive, world-wide license to publish the Supplemental Material files. Please contact the corresponding author directly for reuse.

## References

[1] Zhanel GG, Golden AR, Zelenitsky S, Wiebe K, Lawrence CK, Adam HJ, Idowu T, Domalaon R, Schweizer F, Zhanel MA, Lagacé-Wiens PRS, Walkty AJ, Noreddin A, Lynch Iii JP, Karlowsky JA. 2019. Cefiderocol: a siderophore cephalosporin with activity against carbapenem-resistant and multidrug-resistant Gram-negative Bacilli. Drugs 79:271–289. doi:10.1007/s40265-019-1055-2 30712199

[2] Sato T, Yamawaki K. 2019. Cefiderocol: discovery, chemistry, and in vivo profiles of a novel siderophore cephalosporin. Clin Infect Dis 69:S538–S543. doi:10.1093/cid/ciz826 31724047 PMC6853759

[3] Ito A, Sato T, Ota M, Takemura M, Nishikawa T, Toba S, Kohira N, Miyagawa S, Ishibashi N, Matsumoto S, Nakamura R, Tsuji M, Yamano Y. 2018. In vitro antibacterial properties of cefiderocol, a novel siderophore cephalosporin, against Gram-negative bacteria. Antimicrob Agents Chemother 62:e01454-17. doi:10.1128/AAC.01454-17 PMC5740388 29061741

[4] Jacobs MR, Abdelhamed AM, Good CE, Rhoads DD, Hujer KM, Hujer AM, Domitrovic TN, Rudin SD, Richter SS, van Duin D, Kreiswirth BN, Greco C, Fouts DE, Bonomo RA. 2019. ARGONAUT-I: activity of cefiderocol (S-649266), a siderophore cephalosporin, against gram-negative bacteria, including carbapenem-resistant nonfermenters and Enterobacteriaceae with defined extended-spectrum β-Lactamases and carbapenemases. Antimicrob Agents Chemother 63:e01801-18. doi:10.1128/AAC.01801-18 30323050 PMC6325197

[5] Ito A, Nishikawa T, Ota M, Ito-Horiyama T, Ishibashi N, Sato T, Tsuji M, Yamano Y. 2018. Stability and low induction propensity of cefiderocol against chromosomal AmpC β-lactamases of Pseudomonas aeruginosa and Enterobacter cloacae. J Antimicrob Chemother 73:3049–3052. doi:10.1093/jac/dky317 30188999 PMC6198743

[6] Campanella E, Marino A, Stracquadanio S, Restivo R, Micali C, Nunnari G, Cacopardo B, Ceccarelli M. 2023. Management of ventilator-associated pneumonia due to Stenotrophomonas maltophilia infection: a case report and literature review. World Acad Sci J 5:16. doi:10.3892/wasj.2023.193

[7] Stracquadanio S, Torti E, Longshaw C, Henriksen AS, Stefani S. 2021. In vitro activity of cefiderocol and comparators against isolates of Gram-negative pathogens from a range of infection sources: SIDERO-WT-2014-2018 studies in Italy. J Glob Antimicrob Resist 25:390–398. doi:10.1016/j.jgar.2021.04.019 34020073

[8] 2017. Prioritization of pathogens to guide discovery, research and development of new antibiotics for drug-resistant bacterial infections, including tuberculosis. World Health Organization, Geneva.

[9] Ito A, Nishikawa T, Matsumoto S, Yoshizawa H, Sato T, Nakamura R, Tsuji M, Yamano Y. 2016. Siderophore cephalosporin cefiderocol utilizes ferric iron transporter systems for antibacterial activity against Pseudomonas aeruginosa. Antimicrob Agents Chemother 60:7396–7401. doi:10.1128/AAC.01405-16 27736756 PMC5119021

[10] Karakonstantis S, Rousaki M, Kritsotakis EI. 2022. Cefiderocol: systematic review of mechanisms of resistance, heteroresistance and in vivo emergence of resistance. Antibiotics (Basel) 11:723. doi:10.3390/antibiotics11060723 35740130 PMC9220290

[11] Sheldon JR, Skaar EP. 2020. Acinetobacter baumannii can use multiple siderophores for iron acquisition, but only acinetobactin is required for virulence. PLoS Pathog 16:e1008995. doi:10.1371/journal.ppat.1008995 33075115 PMC7595644

[12] Escalante J, Nishimura B, Tuttobene MR, Subils T, Mezcord V, Actis LA, Tolmasky ME, Bonomo RA, Ramirez MS. 2023. The iron content of human serum albumin modulates the susceptibility of Acinetobacter baumannii to cefiderocol. Biomedicines 11:639. doi:10.3390/biomedicines11020639 36831178 PMC9953112

[13] Moynié L, Luscher A, Rolo D, Pletzer D, Tortajada A, Weingart H, Braun Y, Page MGP, Naismith JH, Köhler T. 2017. Structure and function of the PiuA and PirA siderophore-drug receptors from Pseudomonas aeruginosa and Acinetobacter baumannii. Antimicrob Agents Chemother 61:e02531-16. doi:10.1128/AAC.02531-16 28137795 PMC5365723

[14] He Y, Wang Y, Ma X, Zhao L, Guan J, Zhao J, Yu W, Li Y, Ni W, Gao Z. 2022. Resistance to cefiderocol involved expression of PER-1 β-Lactamase and downregulation of iron transporter system in carbapenem-resistant Acinetobacter baumannii. Infect Drug Resist 15:7177–7187. doi:10.2147/IDR.S392241 36514799 PMC9741825

[15] European Committee on Antimicrobial Susceptibility Testing. 2020. Break- points for cefiderocol from EUCAST: addendum (May 2020) to EUCAST break- point tables v. 10.0: breakpoints to be included in EUCAST breakpoint tables v. 11.0, January 2021

[16] Aung MS, Hlaing MS, San N, Aung MT, Mar TT, Kobayashi N. 2021. Clonal diversity of Acinetobacter baumannii clinical isolates in Myanmar: identification of novel ST1407 harboring blaNDM-1. New Microbes New Infect 40:100847. doi:10.1016/j.nmni.2021.100847 33732472 PMC7944022

[17] Cayô R, Rodríguez M-C, Espinal P, Fernández-Cuenca F, Ocampo-Sosa AA, Pascual A, Ayala JA, Vila J, Martínez-Martínez L. 2011. Analysis of genes encoding penicillin-binding proteins in clinical isolates of Acinetobacter baumannii. Antimicrob Agents Chemother 55:5907–5913. doi:10.1128/AAC.00459-11 21947403 PMC3232777

[18] Wong TY, Hall JM, Nowak ES, Boehm DT, Gonyar LA, Hewlett EL, Eby JC, Barbier M, Damron FH. 2019. Analysis of the in vivo transcriptome of bordetella pertussis during infection of mice. mSphere 4:e00154-19. doi:10.1128/mSphereDirect.00154-19 30996109 PMC6470212

[19] Damron FH, Oglesby-Sherrouse AG, Wilks A, Barbier M. 2016. Dual-seq transcriptomics reveals the battle for iron during Pseudomonas aeruginosa acute murine pneumonia. Sci Rep 6:39172. doi:10.1038/srep39172 27982111 PMC5159919

[20] Kumar SS, Tandberg JI, Penesyan A, Elbourne LDH, Suarez-Bosche N, Don E, Skadberg E, Fenaroli F, Cole N, Winther-Larsen HC, Paulsen IT. 2018. Dual transcriptomics of host-pathogen interaction of cystic fibrosis isolate Pseudomonas aeruginosa PASS1 with Zebrafish. Front Cell Infect Microbiol 8:406. doi:10.3389/fcimb.2018.00406 30524971 PMC6262203

[21] Cafiso V, Stracquadanio S, Lo Verde F, Gabriele G, Mezzatesta ML, Caio C, Pigola G, Ferro A, Stefani S. 2018. Colistin resistant A. baumannii: genomic and transcriptomic traits acquired under colistin therapy. Front Microbiol 9:3195. doi:10.3389/fmicb.2018.03195 30666237 PMC6330354

[22] Cafiso V, Stracquadanio S, Lo Verde F, De Guidi I, Zega A, Pigola G, Stefani S. 2020. Genomic and long-term transcriptomic imprints related to the daptomycin mechanism of action occurring in daptomycin- and methicillin-resistant Staphylococcus aureus under daptomycin exposure. Front Microbiol 11:1893. doi:10.3389/fmicb.2020.01893 32922373 PMC7456847

[23] Allen JL, Tomlinson BR, Casella LG, Shaw LN. 2020. Regulatory networks important for survival of Acinetobacter baumannii within the host. Curr Opin Microbiol 55:74–80. doi:10.1016/j.mib.2020.03.001 32388085 PMC7311227

[24] Kubanova AA, Kubanov AA, Kozhushnaya OS, Vorob’ev DV, Solomka VS, Frigo NV. 2014. The role of some individual amino acid substitutions in penicillin-binding protein (PBP2) of Neisseria gonorrhoeae in the emergence of resistance to ceftriaxone. Mol Biol 48:858–863. doi:10.1134/S0026893314060119 25845238

[25] Nordmann P, Shields RK, Doi Y, Takemura M, Echols R, Matsunaga Y, Yamano Y. 2022. Mechanisms of reduced susceptibility to cefiderocol among isolates from the CREDIBLE-CR and APEKS-NP clinical trials. Microb Drug Resist 28:398–407. doi:10.1089/mdr.2021.0180 35076335 PMC9058874

[26] Price TK, Davar K, Contreras D, Ward KW, Garner OB, Simner PJ, Yang S, Chandrasekaran S. 2022. Case report and genomic analysis of cefiderocol-resistant Escherichia coli clinical isolates. Am J Clin Pathol 157:257–265. doi:10.1093/ajcp/aqab115 34542575

[27] Wienholtz NH, Barut A, Nørskov-Lauritsen N. 2017. Substitutions in PBP3 confer resistance to both ampicillin and extended-spectrum cephalosporins in Haemophilus parainfluenzae as revealed by site-directed mutagenesis and gene recombinants. J Antimicrob Chemother 72:2544–2547. doi:10.1093/jac/dkx157 28582518

[28] Esterly JS, Richardson CL, Eltoukhy NS, Qi C, Scheetz MH. 2011. Genetic mechanisms of antimicrobial resistance of Acinetobacter baumannii. Ann Pharmacother 45:218–228. doi:10.1345/aph.1P084 21304033

[29] Li H, Luo Y-F, Williams BJ, Blackwell TS, Xie C-M. 2012. Structure and function of OprD protein in Pseudomonas aeruginosa: from antibiotic resistance to novel therapies. Int J Med Microbiol 302:63–68. doi:10.1016/j.ijmm.2011.10.001 22226846 PMC3831278

[30] Berrazeg M, Jeannot K, Ntsogo Enguéné VY, Broutin I, Loeffert S, Fournier D, Plésiat P. 2015. Mutations in β-Lactamase AmpC increase resistance of Pseudomonas aeruginosa isolates to antipseudomonal cephalosporins. Antimicrob Agents Chemother 59:6248–6255. doi:10.1128/AAC.00825-15 26248364 PMC4576058

[31] Kawai A, McElheny CL, Iovleva A, Kline EG, Sluis-Cremer N, Shields RK, Doi Y. 2020. Structural basis of reduced susceptibility to ceftazidime-avibactam and cefiderocol in Enterobacter cloacae due to AmpC R2 loop deletion. Antimicrob Agents Chemother 64:e00198-20. doi:10.1128/AAC.00198-20 32284381 PMC7318025

[32] Cain TJ, Smith AT. 2021. Ferric iron reductases and their contribution to unicellular ferrous iron uptake. J Inorg Biochem 218:111407. doi:10.1016/j.jinorgbio.2021.111407 33684686 PMC8035299

[33] Simner PJ, Patel R. 2020. Cefiderocol antimicrobial susceptibility testing considerations: the Achilles' heel of the trojan horse? J Clin Microbiol 59:e00951-20. doi:10.1128/JCM.00951-20 32727829 PMC7771437

[34] Matuschek E, Longshaw C, Takemura M, Yamano Y, Kahlmeter G. 2022. Cefiderocol: EUCAST criteria for disc diffusion and broth microdilution for antimicrobial susceptibility testing. J Antimicrob Chemother 77:1662–1669. doi:10.1093/jac/dkac080 35289853 PMC9155621

[35] Hackel MA, Tsuji M, Yamano Y, Echols R, Karlowsky JA, Sahm DF. 2019. Reproducibility of broth microdilution MICs for the novel siderophore cephalosporin, cefiderocol, determined using iron-depleted cation-adjusted Mueller-Hinton broth. Diagn Microbiol Infect Dis 94:321–325. doi:10.1016/j.diagmicrobio.2019.03.003 31029489

[36] Stracquadanio S, Bonomo C, Marino A, Bongiorno D, Privitera GF, Bivona DA, Mirabile A, Bonacci PG, Stefani S. 2022. Acinetobacter baumannii and cefiderocol, between cidality and adaptability. Microbiol Spectr 10:e0234722. doi:10.1128/spectrum.02347-22 36173300 PMC9603721

[37] Illumina. 2020. Illumina DNA prep reference guide, version 9. Document 1000000025416 v09. San Diego, CA Illumina. https://support.illumina.com/content/dam/illumina-support/documents/documentation/chemistry_documentation/illumina_prep/illumina-dna-prep-reference-guide-1000000025416-09.pdf.

[38] Illumina. 2022. Local run manager v3 software guide. San Diego, CA Illumina. https://support.illumina.com/content/dam/illumina-support/documents/documentation/software_documentation/local-run-manager/local-run-manager.

[39] Kim D, Paggi JM, Park C, Bennett C, Salzberg SL. 2019. Graph-based genome alignment and genotyping with HISAT2 and HISAT-genotype. Nat Biotechnol 37:907–915. doi:10.1038/s41587-019-0201-4 31375807 PMC7605509

[40] Tarazona S, Furió-Tarí P, Turrà D, Pietro AD, Nueda MJ, Ferrer A, Conesa A. 2015. Data quality aware analysis of differential expression in RNA-seq with NOISeq R/Bioc package. Nucleic Acids Res 43:e140. doi:10.1093/nar/gkv711 26184878 PMC4666377

[41] Gu Z, Eils R, Schlesner M. 2016. Complex heatmaps reveal patterns and correlations in multidimensional genomic data. Bioinformatics 32:2847–2849. doi:10.1093/bioinformatics/btw313 27207943

[42] Wei T, Simko V. 2021. R package 'corrplot': visualization of a correlation matrix. Version 0.92. https://github.com/taiyun/corrplot.

[43] Kassambara A. 2023. ggpubr: 'ggplot2' based publication ready plots. R package version 0.6.0. https://CRAN.R-project.org/package=ggpubr.

[44] NCBI Resource Coordinators. 2016. Database resources of the national center for biotechnology information. Nucleic Acids Res 44:D7–D19. doi:10.1093/nar/gkv1290 26615191 PMC4702911

[45] Xavier BB, Mysara M, Bolzan M, Ribeiro-Gonçalves B, Alako BTF, Harrison P, Lammens C, Kumar-Singh S, Goossens H, Carriço JA, Cochrane G, Malhotra-Kumar S. 2020. BacPipe: a rapid, user-friendly whole-genome sequencing pipeline for clinical diagnostic bacteriology. iScience 23:100769. doi:10.1016/j.isci.2019.100769 31887656 PMC6941874

[46] Li H, Durbin R. 2009. Fast and accurate short read alignment with Burrows-Wheeler transform. Bioinformatics 25:1754–1760. doi:10.1093/bioinformatics/btp324 19451168 PMC2705234

[47] Li H. 2011. A statistical framework for SNP calling, mutation discovery, association mapping and population genetical parameter estimation from sequencing data. Bioinformatics 27:2987–2993. doi:10.1093/bioinformatics/btr509 21903627 PMC3198575

[48] Danecek P, Bonfield JK, Liddle J, Marshall J, Ohan V, Pollard MO, Whitwham A, Keane T, McCarthy SA, Davies RM, Li H. 2021. Twelve years of SAMtools and BCFtools. Gigascience 10:giab008. doi:10.1093/gigascience/giab008 33590861 PMC7931819

[49] Rice P, Longden I, Bleasby A. 2000. EMBOSS: the European molecular biology open software suite. Trends Genet 16:276–277. doi:10.1016/s0168-9525(00)02024-2 10827456

[50] Madden T. 2002. The BLAST sequence analysis tool. In McEntyre J, J Ostell (ed), The NCBI Handbook. National Center for Biotechnology Information (US), Bethesda (MD). http://www.ncbi.nlm.nih.gov/books/NBK21097.

[51] Alcock BP, Huynh W, Chalil R, Smith KW, Raphenya AR, Wlodarski MA, Edalatmand A, Petkau A, Syed SA, Tsang KK, et al.. 2023. CARD 2023: expanded curation, support for machine learning, and resistome prediction at the Comprehensive Antibiotic Resistance Database. Nucleic Acids Res 51:D690–D699. doi:10.1093/nar/gkac920 36263822 PMC9825576

[52] Florensa AF, Kaas RS, Clausen P, Aytan-Aktug D, Aarestrup FM. 2022. ResFinder - an open online resource for identification of antimicrobial resistance genes in next-generation sequencing data and prediction of phenotypes from genotypes. Microb Genom 8:000748. doi:10.1099/mgen.0.000748 35072601 PMC8914360

[53] Feldgarden M, Brover V, Haft DH, Prasad AB, Slotta DJ, Tolstoy I, Tyson GH, Zhao S, Hsu C-H, McDermott PF, Tadesse DA, Morales C, Simmons M, Tillman G, Wasilenko J, Folster JP, Klimke W. 2019. Validating the AMRFinder tool and resistance gene database by using antimicrobial resistance genotype-phenotype correlations in a collection of isolates. Antimicrob Agents Chemother 63:e00483-19. doi:10.1128/AAC.00483-19 31427293 PMC6811410

[54] Jolley KA, Bray JE, Maiden MCJ. 2018. Open-access bacterial population genomics: BIGSdb software, the PubMLST.org website and their applications. Wellcome Open Res 3:124. doi:10.12688/wellcomeopenres.14826.1 30345391 PMC6192448

