# Supplementary material for: Role of transcriptomic and genomic analyses in improving the comprehension of cefiderocol activity in Acinetobacter baumannii
Source: mSphere. 2023 Dec 11;9(1):e00617-23. doi: 10.1128/msphere.00617-23 (PMC10826366; doi:10.1128/msphere.00617-23)
Supplement: Legends — for the supplemental materials. [file msphere.00617-23-s0001.docx]

Table S1. Amino acid changes in proposed determinant of FDC resistance (Ref. genome *Acinetobacter baumannii* K09-14 NZ_CP043953.1)

Table S2. Correlation between MIC and gene expressions in CAMHB

ID indicates the locus tag of the gene in *Acinetobacter baumannii* K09-14 NZ_CP043953.1 reference genome.

Only correlations with a significative p-value (≤0.5) are reported in the table.

Table S3. Correlation between MIC and gene expressions in IDCAMHB

ID indicates the locus tag of the gene in *Acinetobacter baumannii* K09-14 NZ_CP043953.1 reference genome.

Only correlations with a significative p-value (≤0.5) are reported in the table.
