# Supplementary material for: Role of transcriptomic and genomic analyses in improving the comprehension of cefiderocol activity in Acinetobacter baumannii
Source: mSphere. 2023 Dec 11;9(1):e00617-23. doi: 10.1128/msphere.00617-23 (PMC10826366; doi:10.1128/msphere.00617-23)
Supplement: Table S1 — Amino acid changes in proposed determinant of FDC resistance. [file msphere.00617-23-s0002.docx]

Table S1. Amino acid changes in proposed determinant of FDC resistance (Ref. genome *Acinetobacter baumannii* K09-14 NZ_CP043953.1)

| Protein (locus ID) | Abau1 | Abau2 | Abau3 | Abau4 | ACICU | ATCC 19606 | ATCC 17978 |
| --- | --- | --- | --- | --- | --- | --- | --- |
| PBP1a (F3P16_RS01455) |  |  |  |  | L147I | V623I |  |
| PBP1b (F3P16_RS06330) | P112S  I460V S764P | P112S I460V S764P | P112S I460V S764P | P112S I460V S764P | P112S  I460V S764P | V110I  I460V  M726V S764P | I460V  S764P |
| PBP2 (F3P16_RS13680) | L424F |  |  |  |  |  |  |
| PBP3 (F3P16_RS01425) |  | A514V |  |  | A346V  H370Y |  |  |
| OprD (F3P16_RS17620) |  | L47I T48S |  | L47I T48S |  |  | L47l  T48S  Q53K  K56N  T58N  F61V  S64T  S68N  E70D  Q101N  K105Q  G120A  S121N  G148A  M150L  N167D  D178N  T307S  V310I  D313N  F321G  I324V  M331L  H338D  N347S  V348L  F365Y  V376L  R383S |
| OprD (F3P16_RS14255) | E25Q  K41E  Q60H  H138Q  V214L  T215N  L293I  E316A  V320I  T341S  K417Q  H445R | G11S  G13S  K41E  M74L  T115A  L116F  I121L  T215N  W243* | E25Q  K41E  Q60H  H138Q  V214L  T215N  L293I  E316A  V320I  T341S  K417Q  H445R | G11S  G13S  K41E  M74L  T115A  L116F  I121L  T215N  W243* | K41E  L116F  K128Q  H138Q  T215N  L238M  L293I  V299I  E316D  D434Y  H445R | G13S  K41E  M74L  H138Q  T215N  L236F  E316A  V320I  V348I  F418I  H445R | K41E  M74I  I172M  V214L  T215N  V384I  V388L  E316A  D434G  H445R |
| OprD (F3P16_RS12500) | A15T  F125Y  I143V  H219R  T381A  F386L | A15T  F125Y  I143V  H219R | A15T  F125Y  I143V  H219R | A15T  F125Y  I143V  H219R | A15T  F125Y  I143V  H219R | I143V  H219R | F3L  A15T  H219R  T381A |
| OprD (F3P16_RS08550) | F14V  R25W  P33T  F176L  S201A  K252N  I284V  I374V  F384L | F14V  R25W  P33T  F176L  S201A  K252N  I284V  I374V | F14V  R25W  P33T  F176L  S201A  K252N  I284V  I374V | F14V  R25W  P33T  F176L  S201A  K252N  I284V  I374V | F14V  R25W  P33T  F176L  S201A  K252N  I284V  I374V | R25W  P33T  F176L  S201A  K252N  I284V  I374V  A379T | F14I  F18L  I19L  S23N  R25W  P33T  F176L  K252N  I284V  I374V |
| AmpC (F3P16_RS05680) | V119E  Q150K  P167S  P238R  F283R | V119E  Q150K  P167S  G247S  F283R  N341T | V119E  Q150K  P167S  P238R  F283R | V119E  Q150K  P167S  F283R  N341T | S80R  V119E  K163Q  G183R  N311S  N379D | V119E  A270T  F283R |  |
